# Fluticasone Propionate/Anti-IgE Combination Preserves Bone Mechanical and Mineral Integrity Better than Monotherapies or Anti-TNF-α in Mice with Ovalbumin-Induced Allergic Airway Inflammation

**DOI:** 10.3390/ijms27125283

**Published:** 2026-06-10

**Authors:** Serkan Gürgül, Can Demirel, Yahya Nural, Özlem Keskin, Mehmet Yaşar Özkars

**Affiliations:** 1 Department of Biophysics, Faculty of Medicine, Gaziantep University, TR-27310 Gaziantep, Turkey; cdemirel27@gmail.com; 2 Department of Analytical Chemistry, Faculty of Pharmacy, Mersin University, TR-33169 Mersin, Turkey; ynural1805@yahoo.com; 3 Department of Pediatric Allergy & Immunology, Faculty of Medicine, Gaziantep University, TR-27310 Gaziantep, Turkey; okeskin02@yahoo.com; 4 Department of Child Health & Diseases, Faculty of Medicine, Kahramanmaraş Sutcu Imam University, TR-46040 Kahramanmaraş, Turkey; myozkars@hotmail.com

**Keywords:** anti-IgE, anti-TNF, fluticasone propionate, chronic allergic airway inflammation, bone mechanical integrity, mineral integrity, inductively coupled plasma-mass spectrometry

## Abstract

Allergic asthma is among the type 2-driven chronic inflammatory allergic diseases. Osteoimmunological findings indicate that shared systemic immune mediators and persistent inflammation can disrupt skeletal homeostasis and promote bone fragility. Treatment commonly includes inhaled corticosteroids like fluticasone propionate (FP) to suppress inflammation, with anti-immunoglobulin E (anti-IgE) biologics added to interrupt the IgE-mediated allergic cascade. TNF-α inhibitors (anti-TNF) are also being studied for their impact on inflammatory pathways. However, their capacity to preserve bone mechanical/mineral integrity in allergic airway inflammation (AAI) remains unclear. This study compared the efficacy of anti-TNF, FP, and anti-IgE monotherapies and an FP/anti-IgE combination in mitigating AAI-induced deficits in bone mechanical/mineral integrity in an ovalbumin (OVA)-induced chronic murine AAI model. Fifty-six male BALB/c mice (8–10 weeks old; 22–24 g) were randomly assigned to control, AAI (OVA-sensitized/challenged), and five treatment cohorts: FP (2000 μg), low-/high-dose anti-IgE (aIgE-L/aIgE-H; 100/200 μg), anti-TNF (aTNF; 6.25 mg/kg-bw), and FP/aIgE-H combination. Following an 8-week protocol, three-point bending and inductively coupled plasma-mass spectrometry (ICP-MS) were used to assess bone mechanical properties and physicochemical characteristics of the mineral phase (calcium (Ca) and phosphorus (P) levels; stoichiometric Ca/P ratio), respectively. Analyses showed that aIgE-L and FP monotherapies failed to mitigate AAI-induced bone changes. aTNF and aIgE-H monotherapies provided comparable protection of cross-sectional properties, rigidity, energy-to-fracture, elastic modulus, and yield/ultimate moments; however, aIgE-H was more efficacious in preserving Ca and P levels and the stoichiometric Ca/P ratio. The FP/aIgE-H combination demonstrated the greatest efficacy in preventing mechanical deterioration and preserving mineral integrity, suggesting it as the optimal strategy for maintaining skeletal health in the management of type 2-driven AAI.

## 1. Introduction

Asthma is a chronic inflammatory disease with diverse clinical phenotypes, severities, and trajectories, all arising from the interplay of multiple cell types, mediators, and pathophysiological mechanisms [1]. Traditionally, asthma was regarded as a localized inflammatory disease restricted to the conducting airways. However, emerging evidence supports the view of asthma as a systemic disorder, given its association with comorbid cognitive, psychiatric, metabolic, and cardiovascular impairments [2]. Allergic asthma constitutes a distinct phenotype characterized by airway hyperresponsiveness/remodeling, increased production of allergen-specific immunoglobulin E (IgE), and a type 2 (Th2)-polarized immune response with eosinophilic airway inflammation [3]. Although the inflammatory response in allergic asthma primarily originates in the respiratory tract, chronic airway inflammation and sustained Th2 immune responses are associated with systemic inflammatory and metabolic dysregulation, adversely affecting extra-pulmonary organs [2,3,4,5,6]. Both clinical and mechanistic evidence increasingly connect asthma and allergic diseases to impaired skeletal integrity and reduced bone mineral density (BMD) through shared inflammatory and osteoimmunological pathways [4,5,6,7].

The existence of a direct causal relationship between asthma and bone loss remains under debate. Cross-sectional studies have reported reduced spinal BMD and an increased risk of osteopenia/osteoporosis in certain groups of asthmatic patients, even after adjusting for confounders, although causality remains unclear [7]. In contrast, large-scale studies and meta-analyses indicate that the heightened risk of bone fragility is primarily due to cumulative oral corticosteroid (OCS) exposure rather than asthma itself [8,9]. Research on inhaled corticosteroids (ICSs) suggests a dose-dependent risk of impaired bone health, particularly among high-dose and long-term users, although several large studies report minimal or no effect [9,10,11,12]. Given these prevailing clinical uncertainties, the “lung–bone axis” hypothesis, rooted in osteoimmunology, provides a compelling framework by proposing that pulmonary inflammatory signals can directly impair skeletal homeostasis [4,13,14]. Accordingly, sustained airway inflammation may contribute to a systemic pro-inflammatory milieu, wherein cytokines such as tumor necrosis factor-α (TNF-α), interleukin (IL)-6, and IL-1β can modulate the receptor activator of nuclear factor kappa-B (RANK) ligand (RANKL)/osteoprotegerin (OPG)-dependent bone remodeling pathways [4,15,16]. Once released into circulation, these mediators—particularly TNF-α—are thought to promote RANKL-driven osteoclastogenesis and alter the RANKL/OPG balance within the RANK/RANKL/OPG pathway, which is central to bone remodeling [4,15,16,17,18]. TNF-α functions as a potent pro-resorptive factor by enhancing RANKL expression on osteoblast lineage (and other stromal) cells, thereby promoting osteoclastogenesis and shifting the RANKL/OPG balance toward bone resorption within the RANK/RANKL/OPG pathway [4,15,16,17]. This cytokine-driven dysregulation of the RANKL/OPG axis [16], together with TNF-α-induced osteoclast differentiation and inhibition of osteoblast maturation [19,20], culminates in excessive bone resorption, deteriorated bone microarchitecture and porosity, and diminished bone strength [13,16].

Although the lung–bone axis hypothesis is supported by substantial evidence [4,13,14], the relative contributions of asthma-related inflammation and the potential adverse effects of standard OCS/ICS therapies on skeletal health remain limited and partly conflicting, rather than supporting a clear, unified clinical view [5,9,16]. A key rationale for investigating the lung–bone axis is that in chronic allergic airway inflammation (AAI), IgE-mediated mast cell activation and the release of mediators such as TNF-α may not only drive chronic Th2-type airway responses but also, through pathways like RANKL/RANK signaling, promote bone resorption [4,21,22,23,24,25,26]. This supports the concept of a mechanistic link between AAI and bone health, reflecting a complex network of relationships rather than a single canonical axis, as a definitive direct pathway has not yet been established [4,21,22,23,24,25,26].

Therapeutic interventions in allergic asthma management typically rely on ICS-containing regimens, including fluticasone propionate (FP), to suppress airway inflammation [1]. Anti-IgE therapy is not routinely combined with ICSs for most patients but is reserved as an add-on biologic to target the IgE-mediated immune cascade in selected allergic asthma phenotypes that remain uncontrolled despite optimized ICS-based therapy [1]. TNF-α inhibitors (anti-TNF) have been shown to reduce systemic bone resorption and improve bone turnover in inflammatory diseases such as Crohn’s disease and rheumatoid arthritis, with well-established clinical benefits [27,28,29]. Accordingly, cytokine-targeted pathways are being examined as potential steroid-sparing strategies for the management of allergic diseases, including asthma [30]. Within this context, we conducted a comparative analysis of the efficacy of FP, anti-TNF (aTNF), and anti-IgE (low-/high-dose; aIgE-L/aIgE-H) monotherapies, as well as an FP/anti-IgE combination (FP/aIgE-H), in mitigating AAI-induced deficits in skeletal integrity—specifically, bone mechanical properties and mineral composition/stoichiometry. Our objective was to clarify the relative effectiveness of these targeted and standard interventions in preserving skeletal integrity using a BALB/c mouse model of chronic asthma induced by an ovalbumin (OVA) sensitization/challenge protocol. OVA sensitization/challenge in BALB/c mice is a widely accepted murine model of allergic asthma characterized by a robust Th2-polarized immune response [31,32]. This model reliably recapitulates key immunological features of human Th2-type inflammation, including robust Th2 cytokine expression, allergen-specific IgE production, pronounced airway inflammation, and airway hyperresponsiveness/remodeling [31,32]. To our knowledge, this study represents the first direct comparative analysis of these therapeutic interventions on bone integrity within the context of IgE-mediated, Th2-driven AAI.

## 2. Results

### 2.1. Cross-Sectional Geometry

Table 1 summarizes the tibial mid-shaft cross-sectional geometry (CSG) results. No significant differences in span length (L), tibial length (Lo), and aspect ratio (Ar) were observed among the groups. However, the AAI group exhibited significant reductions in both cross-sectional cortical area (CSA) (~15%) and cross-sectional moment of inertia (CSMI) (~28%) compared with the control group (*p* < 0.05). The FP group showed similar but nonsignificant reductions in CSA (~4%) and CSMI (~19%) compared with the control group. CSA and CSMI in the aIgE-L group remained comparable to the control group. Although the aIgE-H (~9% for CSA; ~5% for CSMI) and aTNF (~4% for CSA; ~7% for CSMI) groups showed higher means than the control group, these differences were not significant. Conversely, the aIgE-H group exhibited a significant increase in CSA compared to the AAI (~28%; *p* < 0.001) and FP (~14%; *p* < 0.01) groups. A similar upward trend was observed in the aTNF group, which had increased CSA versus AAI (~21%; *p* < 0.01) and FP (~8%). Notably, the FP/aIgE-H group achieved the highest mean CSA, significantly surpassing the control (~20%), AAI (~40%), FP (~25%), and aIgE-L (~21%) groups (*p* < 0.05 to *p* < 0.001). CSMI properties were similar among the aIgE-H, aTNF, and FP/aIgE-H groups, and each exhibited significant increases in CSMI compared to both AAI (~45%, ~48%, and ~53%, respectively) and FP (~30%, ~33%, and ~37%) (*p* < 0.05 to *p* < 0.001). The section modulus (SM) in the AAI (~28%) and FP (~20%) groups was significantly lower than in the control group (*p* < 0.001). In contrast, the aIgE-L, aIgE-H, aTNF, and FP/aIgE-H groups had significantly higher SM than the AAI (~34–51%) and FP (~22–37%) groups (*p* < 0.001 for all).

### 2.2. Whole-Bone Mechanical Properties

Table 2 presents the whole-bone mechanical findings. Analyses showed that yield moment (MY), ultimate moment (MU), rigidity (R), and energy to failure (EtF) in the AAI (~24%, ~23%, ~16%, and ~38%, respectively), FP (~29%, ~28%, ~16%, and ~39%) and aIgE-L (~28%, ~27%, ~17%, and ~34%) groups were significantly lower than in the control (*p* < 0.01 to *p* < 0.001). The mean values of MY, MU, R, and EtF in the aIgE-H group were significantly higher than those in the AAI (~26%, ~25%, ~19%, and ~73%, respectively), FP (~35%, ~33%, ~18%, and ~75%), and aIgE-L (~33%, ~31%, ~19%, and ~63%) groups (*p* < 0.05 to *p* < 0.001). The aTNF group also exhibited a similar upward trend across all structural properties; however, compared with AAI (~18% and ~69%, respectively), FP (~17% and ~71%), and aIgE-L (~18% and ~60%), the increases reached statistical significance only for R and EtF (*p* < 0.01 for R; *p* < 0.001 for EtF). The FP/aIgE-H group achieved the highest means for MY, MU, R, and EtF, notably surpassing the AAI, FP, and aIgE-L groups by ~41–52% (MY), ~42–51% (MU), ~28–29% (R), and ~66–78% (EtF), respectively (*p* < 0.001 for all). The AAI, FP, and aIgE-L groups displayed nonsignificant reductions in post-yield displacement (PYD) compared with the control group. In contrast, the increase in PYD was significant in the aIgE-H group compared with the FP (~81%; *p* < 0.01) and aIgE-L (~71%; *p* < 0.05) groups, and in the aTNF group compared with the FP (~75%; *p* < 0.05) group.

Table 3 summarizes the one-way analysis of covariance (ANCOVA) results. Consistent with previous findings on MU, these results indicate that the group assignment exerted a significant effect on MU after accounting for CSG. Post hoc analysis revealed that MU in the AAI, FP, and aIgE-L groups was significantly lower than in the control (~40%, ~40%, and ~28%, respectively), aIgE-H (~65%, ~64%, and ~38%), and aTNF (~57%, ~57%, and ~31%) groups (*p* < 0.05 to *p* < 0.0001). After controlling for CSG, the FP/aIgE-H group exhibited significantly higher MU than the AAI (~91%), FP (~90%), and aIgE-L (~59%) groups (*p* < 0.0001 for all), as well as a ~21% increase over the aTNF group (*p* < 0.05).

**Figure 1:**
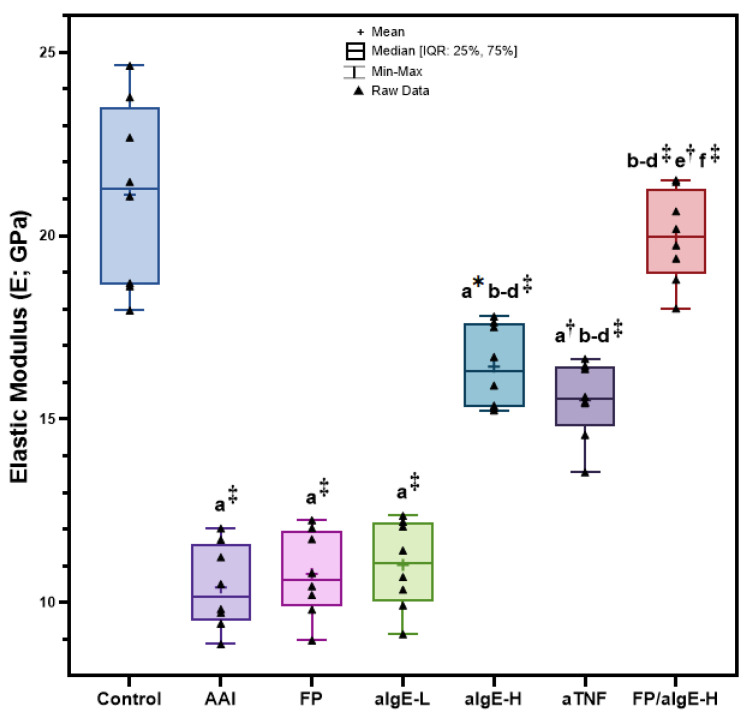
Estimated elastic modulus (E) findings of the groups. One-way analysis of variance (ANOVA) followed by Tamhane T2 post hoc test was used. ^a^ vs. control, ^b^ vs. AAI, ^c^ vs. FP, ^d^ vs. aIgE-L, ^e^ vs. aIgE-H, ^f^ vs. aTNF. * *p* < 0.05, ^†^ *p* < 0.01, ^‡^ *p* < 0.001 [*n* = 8 per group; F (6, 49) = 78.45, *p* = 0.0001, η^2^ = 0.906]. Control (saline); AAI (allergic airway inflammation; OVA-sensitized/challenged); FP (2000 µg fluticasone propionate); aIgE-L (100 µg anti-IgE); aIgE-H (200 µg anti-IgE); aTNF (6.25 mg/kg anti-TNF); FP/aIgE-H (combination of 2000 µg FP/200 µg aIgE). All treatments were administered during the OVA challenge phase.

Figure 1 displays the estimated elastic modulus (E) findings for each group. Compared with the control group, the E means in the AAI (~51%), FP (~49%), and aIgE-L (~48%) groups were significantly reduced (*p* < 0.001 for all). The aIgE-H and aTNF groups had considerably higher E means than the AAI (~58% and ~49%, respectively), FP (~53% and ~44%), and aIgE-L (~49% and ~41%) groups (*p* < 0.001 for all). However, both groups remained significantly below control levels (~22% for aIgE-H, *p* < 0.05; ~27% for aTNF, *p* < 0.01). Notably, the FP/aIgE-H group achieved the highest mean E overall, significantly surpassing the AAI (~92%), FP (~85%), aIgE-L (~81%), aIgE-H (~21%), and aTNF groups (~29%) (*p* < 0.01 to *p* < 0.001).

### 2.3. Bone Mineral Composition and Stoichiometry

**Figure 2:**
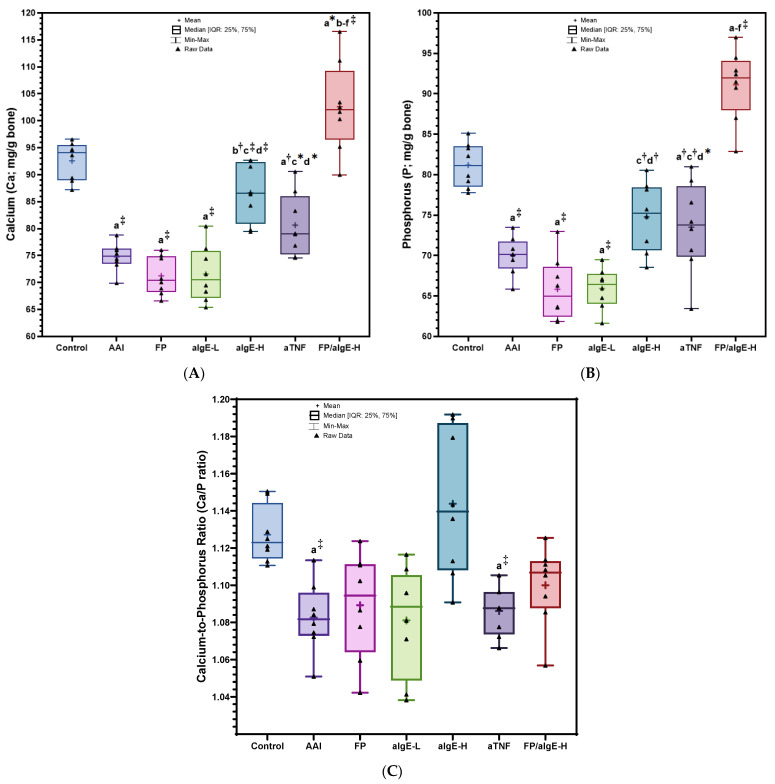
Bone mineral composition and stoichiometry findings of the groups. Calcium (**A**), phosphorus (**B**), and the stoichiometric calcium-to-phosphorus ratio (**C**). One-way ANOVA followed by Fisher’s Least Significant Difference (LSD) pairwise comparisons with Bonferroni correction (for Ca and P) or Tamhane T2 (for the Ca/P ratio) post hoc tests were used. ^a^ vs. control, ^b^ vs. AAI, ^c^ vs. FP, ^d^ vs. aIgE-L, ^e^ vs. aIgE-H, ^f^ vs. aTNF. * *p* < 0.05, ^†^ *p* < 0.01, ^‡^ *p* < 0.001 [*n* = 8 per group; F (6, 49) = 40.96, *p* = 0.0001, η^2^ = 0.833 for Ca; F (6, 49) = 43.80, *p* = 0.0001, η^2^ = 0.844 for P; F (6, 49) = 7.16, *p* = 0.0001, η^2^ = 0.483 for Ca/P-ratio]. Control (saline); AAI (allergic airway inflammation; OVA-sensitized/challenged); FP (2000 µg fluticasone propionate); aIgE-L (100 µg anti-IgE); aIgE-H (200 µg anti-IgE); aTNF (6.25 mg/kg anti-TNF); FP/aIgE-H (combination of 2000 µg FP/200 µg aIgE). All treatments were administered during the OVA challenge phase.

Figure 2A–C present the bone calcium (Ca) and phosphorus (P) levels, as well as the stoichiometric Ca/P ratio. Compared with the control group, Ca and P levels were considerably reduced in the AAI, FP, aIgE-L, and aTNF groups (~13–23% for Ca, ~9–19% for P; *p* < 0.01 to *p* < 0.001; Figure 2A,B). The aIgE-H group exhibited significantly higher Ca levels than the AAI (~16%), FP (~22%), and aIgE-L (~21%) groups and higher P levels than the FP (~14%) and aIgE-L (~13%) groups (*p* < 0.01 to *p* < 0.001; Figure 2A,B). Similarly, the aTNF group exceeded the FP (~13% and ~12%, respectively) and aIgE-L (~13% and ~11%) groups in Ca and P levels (*p* < 0.05 to *p* < 0.01; Figure 2A,B). Remarkably, the FP/aIgE-H group achieved the highest mean Ca and P levels, considerably exceeding all other groups and the control group (~11–44% for Ca, ~12–38% for P; *p* < 0.05 to *p* < 0.001; Figure 2A,B). The stoichiometric Ca/P ratio in the FP, aIgE-L, aIgE-H, and FP/aIgE-H groups remained comparable to the control group. In contrast, the stoichiometric Ca/P ratio was significantly lower in the AAI and aTNF groups than in the control group (~4% and *p* < 0.01 for both; Figure 2C).

## 3. Discussion

Bone biomechanics is grounded in the concept that bone is structurally designed to withstand high load-bearing capacity, with its composition optimized to support this mechanical function [33]. At the whole-bone level, bone strength (BS; defines bone’s load-bearing ability) and stiffness (reflects bone’s capacity to resist) are among the primary indicators of bone response to loading [34]. In whole-bone bending tests, MY (the moment at which deformation becomes plastic) and MU (maximum moment) are indicators of BS, R is a measure of elastic resistance (denotes the degree to which bone withstands deformation under applied moments), and EtF represents overall bending resistance of bone [34,35,36,37]. In the AAI group, MY (~24%), MU (~23%), R (~16%), and EtF (~38%) were significantly reduced compared to the control group (Table 2). Diminished MY and MU indicated that bones in the AAI group have a lower capacity to resist bending, resulting in reduced BS. Data from R and EtF further confirmed that these bones are less resistant to deformation, indicating increased fragility. PYD, reflecting bone ductility (i.e., the bone’s capacity to undergo plastic deformation without fracture) [34], was also ~18% lower in AAI than in the control (Table 2). These data collectively show that AAI impairs bone structural properties, consistent with the previous literature [38]. Bones from the FP and aIgE-L groups showed mechanical properties similar to those of the AAI group, namely, increased fragility and diminished BS and ductility (Table 2). These changes in structural properties provide strong evidence that the aIgE-L and FP monotherapies failed to prevent AAI-induced structural deterioration in bone. In contrast, the aIgE-H, aTNF, or FP/aIgE-H combination therapies led to more effective prevention, indicating favorable improvements in the relevant parameters and, thus, in BS (Table 2).

Bone CSG is a key determinant of BS and R. In bending tests, it is often assessed using CSA, CSMI, and SM, which are geometric indicators of cortical bone integrity [34,36,39,40]. In the AAI group, CSA (~15%), CSMI (~28%), and SM (~28%) were significantly lower than in the control group, indicating reduced cortical structural integrity (Table 1). These results suggest that AAI may impair bone CSG and, therefore, negatively affect BS and R. The changes in CSG parameters in the AAI group are consistent with the observed reductions in BS and R, as well as with our previous report [38]. Compared with the AAI group, increases in bone CSG parameters across the overall treatment groups (Table 1)—particularly in the aTNF, aIgE-H, and FP/aIgE-H groups—can be considered a beneficial contribution of the relevant therapies to cortical integrity and, thus, to BS and R. Aside from these findings, our study also considered SM to evaluate the overall influence of CSG on BS. The SM, which comprises the entire CSG, is directly proportional to MU [41]. Data indicated that the SM was a reliable predictor of BS, explaining ~56% of the variation in MU between groups (Table 3). However, data from regression lines (not shown; R^2^ = 0.457 for control, R^2^ = 0.267 for AAI, R^2^ = 0.785 for FP, R^2^ = 0.627 for aIgE-L, R^2^ = 0.041 for aIgE-H, R^2^ = 0.246 for aTNF, R^2^ = 0.470 for FP/aIgE-H) revealed that the SM did not provide a complete explanation for the variations in BS between groups, suggesting a change in bone at the tissue-level.

Bone mineral composition is a prominent determinant of BS and R [30]. The bone mineral phase consists mainly of hydroxyapatite crystals (Ca_10_(PO_4_)_6_(OH)_2_), with Ca and P as dominant high-atomic-number constituents [42,43,44]. Bone metabolism and mineral homeostasis depend on the relative contents of Ca and P, making them suitable biomarkers for assessing bone health [42]. Ca and P serve as sensitive metrics of bone mineral changes in clinical and research settings [43]. The stoichiometric Ca/P ratio provides a distinct window into bone quality [45], reflecting physicochemical characteristics of the mineral phase, including maturity and crystallinity [44,46]. Dual-Energy X-ray Absorptiometry (DXA) and Micro-Computed Tomography (μ-CT) are established techniques for assessing BMD and structural microarchitecture in rodent models [47,48]. Although these methods effectively quantify morphological and volumetric changes in bone, they are limited in their ability to resolve the precise elemental stoichiometry of the mineral phase. The inductively coupled plasma-mass spectrometry (ICP-MS) addresses this limitation by offering superior sensitivity, lower detection limits, and precise multi-element analysis for absolute mineral quantification. By directly measuring the mineral composition and stoichiometry of the bone matrix, ICP-MS enables the detection of subtle changes in both major and trace elements [49,50,51]. As shown in Figure 2A–C, Ca (~19%), P (~14%), and the stoichiometric Ca/P ratio (~4%) were significantly reduced in the AAI group compared to the control group. Although Ca and P are primary constituents of the mineral phase, most traditional bone analyses focus on Ca detection. In bone disorders such as osteoporosis, a strong correlation between the extent of Ca loss and decreased BMD has been confirmed [45]. Taken together, these physicochemical shifts—encompassing both compositional and stoichiometric alterations—robustly suggest that AAI actively undermines bone mineral and structural integrity, driving progressive demineralization. These findings are consistent with observed reductions in BS and R, as well as with our previous report [38] and recent evidence that OVA-induced allergic asthma induces bone loss in mice [52]. Gao et al. (2025) reported local bone mass reduction in the primary spongiosa of the proximal tibial metaphysis using μ-CT following an acute OVA-sensitization/challenge protocol (14/4 days) [52]. Our study extends these observations by using ICP-MS to quantify absolute mineral composition (bone Ca and P levels) and stoichiometry (the Ca/P ratio) across the entire tibia under chronic conditions (14 days/8 weeks). Our ICP-MS analyses revealed that sustained allergic inflammation impairs the mineral integrity of the entire bone, including the dense diaphyseal cortical shell, and drives demineralization. Therapeutic interventions, however, demonstrated a distinct hierarchy of efficacy. aIgE-L and FP monotherapies failed to arrest AAI-induced mineral damage. Anti-TNF provided only moderate mitigation. aIgE-H and FP/aIgE-H combination therapy effectively preserved mineral integrity (Figure 2A–C), subsequently enhancing BS and R (Table 1 and Table 2). Among all treatments, FP/aIgE-H combination therapy emerged as the most effective strategy for preserving both mineral composition and stoichiometry.

The detrimental effects of AAI on tissue-level bone mechanical properties have been previously documented. One study evaluated estimated material properties—particularly yield stress, ultimate stress, and E—alongside physicochemical parameters, specifically Ca and P levels and the stoichiometric Ca/P ratio [38]. Observed reductions in estimated properties within the AAI group, together with decreases in physicochemical parameters, were interpreted as evidence of compromised bone mineralization [38], since these indices are highly sensitive to fluctuations in mineral composition [53]. Consistent with this association, a profound ~51% reduction in estimated E was observed in the AAI group relative to the control group, mirroring decreases in physicochemical parameters (Figure 1 and Figure 2A–C). These concurrent reductions signify impaired bone mineralization, a conclusion supported by the established relationship between tissue-level mechanical properties and bone mineral composition and integrity [34,37,38,40,53]. Conversely, physicochemical and E data indicate that FP/aIgE-H combination therapy effectively inhibits AAI-induced changes in mineralization (Figure 1 and Figure 2A–C). Additionally, aIgE-H monotherapy provides greater protection against demineralization than aTNF therapy, as evidenced by higher estimated E and physicochemical parameters (Figure 1 and Figure 2A–C).

The technical reliability of the material property estimations, particularly regarding E, is intrinsically linked to the optimization of the experimental geometry used during three-point bending. In three-point bending, the Ar establishes the relative contribution of bending and shear components to bone deflection. It is desired to be over 20 to maximize pure bending; however, acquiring an optimal Ar through murine femora or tibiae is biologically precluded, as bones are not long enough relative to their width [38,54]. To overcome this morphological constraint and maximize the accuracy of the estimated E values, the Ar was optimized by adjusting L to the maximum stable span (12.67–13.52 mm; Table 1). This methodological refinement prioritizes pure bending by minimizing the confounding effects of shear interference [54]. Nonsignificant differences in L and the Ar between groups indicate that the contributions of bending and shear to bone deflection were analogous across all groups (Table 1). Crucially, this geometric optimization aligns our macro-scale estimates with intrinsic material properties; an increase in Ar yields E values highly consistent with those from micro-scale nanoindentation in mice [54]. Reported E values for murine tibiae obtained via nanoindentation range from 11.5 to 29.9 GPa, depending on age and hydration state [55,56,57]. In this context, estimated mean E values for the control (21.28 GPa), aIgE-H (16.31 GPa), aTNF (15.54 GPa), and FP/aIgE-H (19.97 GPa) groups are highly concordant with these literature benchmarks (Figure 1). In contrast, E values in the AAI (10.16 GPa), FP (10.62 GPa), and aIgE-L (11.06 GPa) groups fell below this range (Figure 1). This agreement with micro-scale standards underscores the conclusion that AAI adversely affects bone physicochemical characteristics and thus mineralization. Notably, aIgE-H and aTNF monotherapies yielded comparable E values within the reported physiological range. However, the nominally higher E values observed in the aIgE-H group compared to the aTNF group suggest that the IgE-mediated pathway may play a more prominent role in AAI-induced bone material deterioration (Figure 1). Furthermore, while aIgE-L and FP monotherapies failed to prevent these deficits, the combination of aIgE-H and FP effectively inhibited AAI-induced changes in mineralization. Once integrated with our physicochemical results, these findings validate the superior protective efficacy of FP/aIgE-H combination therapy in preserving bone quality.

A potential mechanism underlying both the detrimental effects of AAI on bone health and the protective efficacy of high-dose anti-IgE involves mast cell activity within bone tissue [38]. In allergic asthma, mast cells are activated when allergens cross-link IgE bound to the high-affinity IgE receptor (FcεRI) [58]. Once activated, these cells release various inflammatory mediators that can promote osteocatabolic processes (e.g., histamine, TNF-α, IL-6) and/or suppress osteoblastic activity (e.g., TNF-α, IL-1) [58]. The involvement of mast cells in bone diseases, including osteoporosis, has been established in various empirical studies [59,60]. Anti-IgE prevents mast cell activation by binding to the Cε3 domain of circulating IgE. By masking this region, it sterically inhibits IgE from docking into FcεRI receptors, thereby disrupting the IgE/allergen–FcεRI axis. This interaction reduces circulating IgE levels, downregulates FcεRI expression on mast cells, and prevents subsequent allergic reactions [61]. Anti-IgE has been shown to reduce IgE levels, airway inflammation, remodeling, and chronic histological changes in a dose-dependent manner, with higher doses having greater effects [62]. Although evidence on the therapeutic effects of anti-IgE on bone mineral and mechanical properties remains limited, a recent study has demonstrated its dose-dependent efficacy, particularly at 200 µg, in preventing AAI-induced reductions in bone strength and quality [38]. In contrast, FP acts by binding the cytoplasmic glucocorticoid receptor-α. It directly reduces the number of diverse inflammatory cells, including mast cells, by turning off multiple activated inflammatory genes encoding various inflammatory mediators (e.g., TNF-α, IL-6) [63,64]. Recent findings have demonstrated that inflammatory mediators, such as C-C and C-X-C chemokines released from FcεRI-activated mast cells, can attenuate the anti-inflammatory action of FP [65]. Therefore, the attenuation of FP activity by mast cell-derived inflammatory mediators and the inhibitory efficacy of aIgE-H on FcεRI-activated mast cells may explain the changes in bone observed in the FP and FP/aIgE-H groups, respectively.

TNF-α is a well-established immunomodulatory and inflammatory cytokine recognized for its critical role in systemic and/or local bone loss by promoting osteoclastic bone resorption and suppressing osteoblastic bone formation [17,20,66]. Therefore, inhibiting TNF-α has the potential to prevent or reverse bone loss in inflammatory bone diseases such as rheumatoid arthritis. Current evidence indicates that anti-TNF agents improve the bone formation-to-resorption ratio and slow bone loss yet do not provide significant benefit in preventing bone loss [27,28,29]. Partial improvements in bone geometric and mechanical properties were observed following aTNF monotherapy (Table 1 and Table 2); however, physicochemical and estimated E data indicate that it failed to prevent bone loss at the material level (Figure 1 and Figure 2A–C). These findings are consistent with the existing literature noting that anti-TNFs primarily slow rather than reverse systemic bone degradation [27,28,29]. In contrast, aIgE-H, especially when combined with FP, was more effective at preventing bone loss at this level (Figure 1 and Figure 2A–C). This superior efficacy likely stems from its upstream action, which silences the cellular source of inflammation. By sterically inhibiting IgE docking and downregulating surface FcεRI expression, anti-IgE prevents the release of both pre-formed and newly synthesized TNF-α, as well as a broader range of mast cell-derived pro-resorptive mediators such as histamine and IL-6, which are not targeted by anti-TNF monotherapy [61].

To date, this study represents the first comparative investigation of the efficacy of targeted therapies—specifically anti-TNF, FP, and anti-IgE (aIgE-L/aIgE-H) monotherapies, as well as FP/aIgE-H combination—in preserving bone mechanical and mineral integrity in an OVA-induced murine AAI model. Despite comprehensive biomechanical and ICP-MS assessments and the compelling evidence for a functional lung–bone axis, several limitations inherent to the study design warrant acknowledgment. First, this study uses an OVA-induced murine model that primarily replicates the IgE-mediated Th2-driven allergic phenotype of asthma. Consequently, these findings may not be generalizable to the full clinical heterogeneity in human asthma, including non-T2-high (T2-low), neutrophilic, obesity-related, or corticosteroid-resistant asthma endotypes. Future studies are needed to determine whether similar skeletal degradation occurs across all human asthma endotypes. Given the limited human data establishing a causal link between asthma and bone loss, this study is best considered a preclinical investigation into the potential skeletal consequences of IgE-mediated, Th2-driven allergic asthma. Second, osteocatabolic inflammatory mediators, bone turnover biomarkers, cortical/trabecular microarchitecture, or histological/histomorphometric bone parameters were not analyzed. Additionally, this study was not extended to the evaluation of intrinsic bone properties, such as cortical porosity, collagen cross-linking, mineral density distribution, or mineral crystal size and crystallinity. Consequently, the findings are restricted to the comparative efficacy of the evaluated therapeutic interventions on bone mineral composition/stoichiometry, geometry, and mechanical properties, without direct experimental assessment of the cellular or molecular mechanisms underlying these changes. Given the widespread clinical use of ICS and anti-IgE therapies, alongside growing interest in TNF-α blockers for asthma management, understanding their multifaceted effects on bone mineral stoichiometry and mechanics remains essential. These findings may inform the development of nuanced treatment strategies that preserve skeletal health in patients with IgE-mediated, Th2-driven AAI.

## 4. Materials and Methods

Bone samples for the present study were obtained from a previous study by Ozkars et al. (2018) [62]. This previous research used an OVA-induced murine model of chronic asthma conducted in accordance with the protocols described by Temelkovski et al. (1998) [62,67]. In the model, asthmatic mice exhibited a pronounced inflammatory and allergic airway profile compared to healthy controls, as evidenced by coordinated changes in bronchoalveolar lavage fluid (BALF) cell composition/density, tissue inflammation scores, and BALF total IgE levels [62]. Specifically, increased percentages of lymphocytes and macrophages indicate marked lymphocytic airway inflammation and enhanced recruitment of inflammatory cells [62]. Conversely, the sharp decrease in bronchial epithelial cell percentage is consistent with epithelial damage or loss in asthmatic airways [62]. BALF cell density scores were higher in asthmatic mice, suggesting intense overall cellular infiltration into the airways [62]. Histologically, an increased peribronchial/perivascular inflammation score demonstrates robust accumulation of inflammatory cuffs around bronchi and vessels, indicating sustained inflammatory stress and ongoing structural remodeling [62]. Morphometric analyses further supported these findings, revealing a clear trend toward thickening of airway smooth muscle (ASM), suggestive of persistent structural changes [62]. In parallel with these cellular and histological changes, elevated BALF total IgE levels, consistent with an allergen-driven contribution, support an IgE-mediated mechanism underlying this amplified airway inflammation and remodeling [62]. Furthermore, compared with healthy controls, asthmatic mice exhibited an apparent shift toward increased BALF IL-4 levels, elevated miRNA-126 expression (a Th2-polarized pro-inflammatory marker), and reduced miRNA-133a expression (a therapeutic marker associated with ASM function and remodeling) [62]. Collectively, the observed changes in BALF metrics, chronic histological profiles, and elevated BALF total IgE levels provide robust internal validation that the model successfully recapitulates key features of IgE-mediated, Th2-driven chronic allergic airway inflammation with structural remodeling. Moreover, the experimental framework of the prior study enabled a comprehensive comparison of multiple interventions, revealing distinct, parameter-specific patterns in both therapeutic and prophylactic efficacy across all evaluated inflammatory and structural metrics. Accordingly, FP emerged as the least effective intervention, proving largely unsuccessful in both prophylactic and therapeutic contexts. In contrast, aIgE-H and aTNF monotherapies yielded the most pronounced improvements across inflammatory, cellular, and structural markers, with aIgE-H demonstrating the highest efficacy in reversing chronic histological changes. From a prophylactic standpoint, while both the FP/aIgE-H combination and aTNF treatments were effective, the FP/aIgE-H therapy provided the optimal baseline balance by delivering superior dual prophylactic and therapeutic outcomes [62].

The previous research [62] also used a tissue-sharing approach to adhere to the 3Rs (reduction); thus, no additional animals were purchased, bred, or sacrificed, and no live-animal interventions were performed specifically in the research. For the present study, left tibiae were harvested immediately following the terminal procedure (lung sample removal) of the prior study [62] to minimize postmortem degradation. The animal preparation, antigen sensitization/challenge, and treatment protocols of the previous study are briefly summarized in Section 4.1.

### 4.1. Animal Preparation, Antigen Sensitization/Challenge, and Treatment Protocol

**Figure 3:**
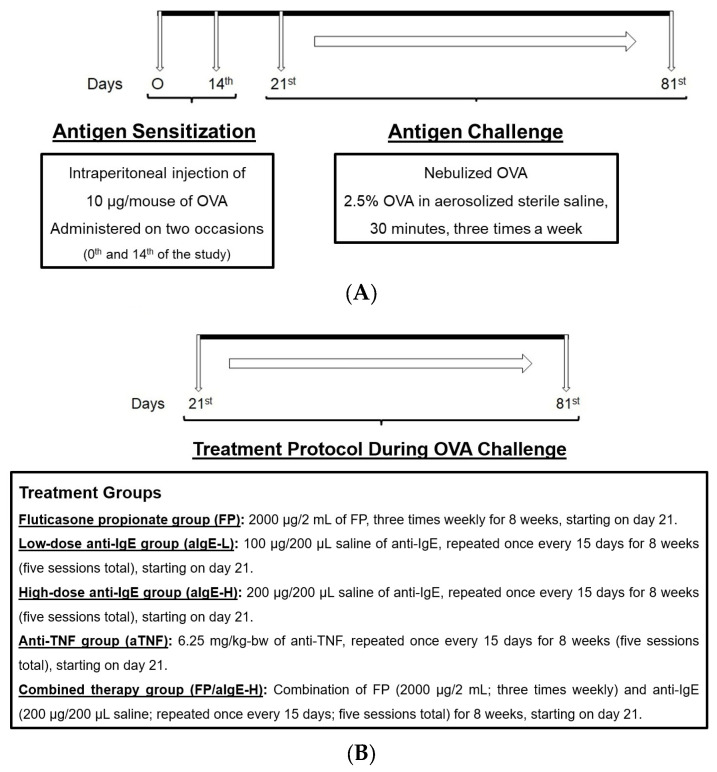
Schematic diagram of the experimental design. Antigen (OVA) sensitization and challenge protocol (**A**). Treatment protocol (**B**).

Fifty-six male BALB/c mice (8–10 weeks old, 22–24 g) were purchased and kept under standard conditions (Appendix A.1). After a 24 h acclimation period without adverse clinical signs, the mice were randomly assigned into seven equal-sized groups (*n* = 8 per group):

1. *Control group*: Neither medication nor antigen was given to this group of mice. The mice were administered saline at the same dose and through the same route as the sensitization and challenge protocol described below (Figure 3A) [38,62].
2. *Chronic AAI* (*allergic airway inflammation*) *group*: The mice were sensitized through intraperitoneal (i.p.) injection of chicken egg OVA (Grade V, Sigma-Aldrich, St. Louis, MO, USA) at doses of 10 μg/200 μL saline (per mouse) on days 0 and 14. Starting on day 21 following immunization, the mice (11–13 weeks old) were challenged with nebulized 2.5% OVA (in sterile saline) using a nebulizer (InnoSpire/Essence, Philips Respironics, Monroeville, PA, USA) for 30 min, three times weekly for 8 weeks (Figure 3A) [38,62].
3. *FP* (*Fluticasone propionate*) *group*: During OVA challenge, the mice received aerosolized 2000 µg/2 mL FP (Flixotide^®^, GSK, Istanbul, Turkey) via a nebulizer, starting on day 21 and repeated three times weekly for 8 weeks (Figure 3B) [62,68,69].
4. *aIgE-L* (*low-dose anti-IgE*) *group*: During OVA challenge, the mice received 100 μg/200 μL saline of anti-IgE mAb (Xolair^®^, Novartis Pharmaceuticals, Istanbul, Turkey) i.p., starting on day 21 and repeated once every 15 days for 8 weeks (a total of five sessions) (Figure 3B) [38,62,70].
5. *aIgE-H* (*high-dose anti-IgE*) *group*: During OVA challenge, the mice received 200 μg/200 μL saline of anti-IgE by the same route and intervals as the aIgE-L group (Figure 3B) [38,62,71].
6. *aTNF* (*anti-TNF*) *group*: During OVA challenge, the mice in this group received 6.25 mg/kg body weight of anti-TNF mAb (Remicade^®^, Pera Pharmaceuticals, Istanbul, Turkey) by the same route and intervals as the aIgE-L/H groups (Figure 3B) [62,72].
7. *FP/aIgE-H combination therapy group*: During OVA challenge, the mice received a combination of FP (2000 µg) and anti-IgE (200 µg) at the same dose, routes, and intervals as the FP and aIgE-H groups (Figure 3B) [62].

Following the final OVA challenge [62], 56 male BALB/c mice (19–21 weeks old) were euthanized by exsanguination via cardiac puncture under deep anesthesia (100 mg/kg ketamine and 12 mg/kg xylazine, i.p.). Before euthanasia, the surgical plane of anesthesia (loss of pedal withdrawal reflex, slowed respiratory rate) was verified. Death was confirmed by the permanent cessation of heartbeat and circulation. After confirmation of death, the left tibia with the surrounding muscles of each mouse was immediately extracted to prevent postmortem degradation and wrapped with saline-moistened gauze pads. Each sample was placed in a standard 15 mL Falcon tube, labeled with alphanumeric codes, and then stored at –20 °C in a standard high-performance laboratory freezer until further analyses were performed (Appendix A.1). Since all tibiae satisfied the predetermined criteria for inclusion, all 56 left tibiae were included in the present study (Appendix A.2).

### 4.2. Cross-Sectional Properties

Bone cross-sectional geometry (CSG) was obtained from the tibial mid-shaft for each bone using a Toshiba Aquilion 64 Slice-CT scan (Toshiba America Medical Systems (TAMS), Tustin, CA, USA) under the following protocols: 120 kVp/150 mA, 0.5 mm slice thickness, a matrix size of 512 × 512 pixels, and an in-plane voxel size of 0.234 mm × 0.234 mm [38]. The region of interest (ROI) encompassed a 1–2 mm segment of the mid-diaphyseal area at the fracture site. Scans provided data for measuring the endosteal (END; mm) and periosteal (PER; mm) diameters in the medio-lateral (ML) and anterior–posterior (AP) planes.

Scans were examined in the axial plane by an investigator blinded to the group identity of each tibia (samples labeled with alphanumeric codes) using Vitrea software (V2.0, Canon Medical Systems USA Inc., Tustin, CA, USA) and were cross-referenced by the same investigator with the point-counting method. To assure precision, readings were replicated 10 times, and the resulting means were then used to compute the cross-sectional area (CSA; mm^2^) and moment of inertia (CSMI; mm^4^) as follows [73]:

(1) $$CSA=\frac{\pi }{4}\cdot \left[\left({PER}_{AP}\cdot {PER}_{ML}\right)-\left({END}_{AP}\cdot {END}_{ML}\right)\right]$$

(2) $$CSMI=\frac{\pi }{64}\cdot \left[\left({PER}_{AP}^{3}\cdot {PER}_{ML}\right)-\left({END}_{AP}^{3}\cdot {END}_{ML}\right)\right]$$

### 4.3. Whole-Bone Mechanical Properties

Bones were tested using a custom-made three-point bending device (MAY-TPBM-2113, Commat Ltd., Ankara, Turkey) equipped with an H3G-6B-50kg-C3 load cell (Zemic Europe BV., Breda, The Netherlands) and a T50-linear potentiometer position sensor (Novotechnik, Southborough, MA, USA), following previously described protocols (Appendix A.1 and Appendix A.2). Tibial length (Lo; mm) was measured using a standard digital caliper. The span length (L; mm), i.e., the distance between the two lower supports, was set to the largest achievable length without compromising bone stability [54]. A vertical load was applied to the tibial mid-diaphysis in the ML plane until failure.

**Figure 4:**
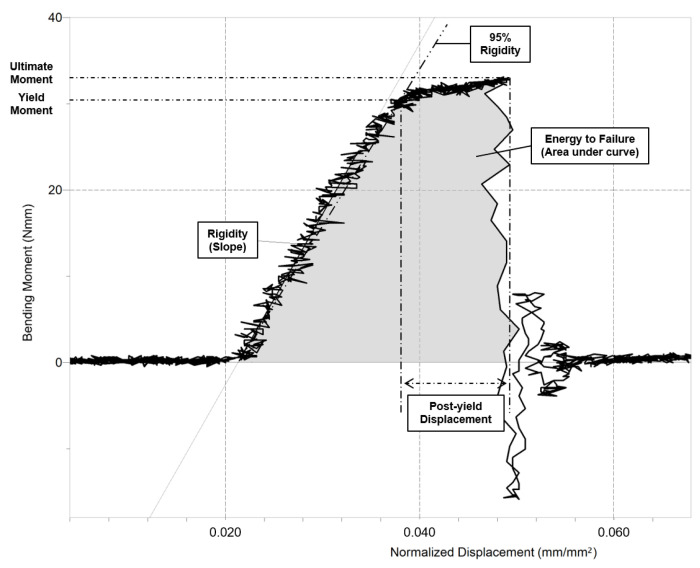
A representative moment vs. normalized displacement curve describing mechanical characteristics of whole bone. Rigidity is the slope of the linear region of the curve. Yield moment is the moment value where the data curve intersects a line with a slope of 95% of the rigidity. Ultimate moment is the maximum moment value. Post-yield displacement is the displacement from the yield point to the point of failure. Energy to failure is the area under the curve [35].

To ascertain whole-bone mechanical characteristics, force (F; N) and displacement (D; mm) data were collected and transformed into moment (M=F·L4; Nmm) and normalized displacement (D′=12·DL2; mm/mm^2^). From the resulting M–D′ curves, the following parameters were determined: MY (yield moment; Nmm), MU (ultimate moment; Nmm), PYD (post-yield displacement; mm/mm^2^), R (rigidity; Nmm^2^), and EtF (energy to failure; N) (Figure 4) [35,36,38]. LoggerPro software (V3.8.3; Vernier, Beaverton, OR, USA) was used to analyze all mechanical data.

In three-point bending, bone deflection mainly results from bending, but shear displacement also occurs. The shear component of the deflection is inversely proportional to the Ar (aspect ratio; Ar=LPERML; mm/mm); hence, Ar determines the relative contributions of bending and shear. Maximizing Ar allows the determination of estimated E (elastic modulus; GPa) consistent with that obtained by nanoindentation in mice [54]. Therefore, E was estimated as follows [74]:

(3) $$E=S\cdot \left(\frac{L^{3}}{48\cdot CSMI}\right)$$

where S denotes the stiffness derived from the initial linear slope of the F − D data.

The SM (section modulus; mm^3^), a geometric measure of a bone’s resistance to bending, is defined as [39,41]:

(4) SM=CSMIc

where c is the half-diameter of mid-shaft (PERML2; mm) in the ML plane. The SM includes all geometric features that affect MU. Any MU differences not explained by the SM are attributed to changes in bone material properties [41].

### 4.4. Bone Mineral Composition and Stoichiometry

The physicochemical characteristics of the bone mineral phase, particularly Ca and P levels and mineral stoichiometry (Ca/P ratio), were assessed to determine the impact of AAI on mineral integrity. To minimize human factor confounders, all procedures were conducted by a primary investigator blinded to the group identity of each tibia using samples labeled with alphanumeric codes; blinding was maintained until the end of data collection and ICP-MS analysis. Ca and P levels were measured using an Agilent 7500ce ICP-MS (Agilent Tech., Tokyo, Japan) equipped with ChemStation built-in software (G1834B) based on established protocols [38,75]. Following the removal of soft tissue, bone samples were weighed to an accuracy of 0.0001 g using a precision scale (Shimadzu-ATX224/Uni Block, Shimadzu Europa GmbH, Duisburg, Germany). For microwave-assisted digestion, the samples were placed in digestion tubes and subjected to a sequential addition of 2 mL 65% nitric acid (~14.34 M; Merck KGaA, Darmstadt, Germany), 6 mL 37% hydrochloric acid (~12.06 M; Merck KGaA), and 1 mL 30% hydrogen peroxide (~9.8 M; Merck KGaA). Once gas evolution ceased, the tubes were inserted into the microwave oven at 160 °C for 10 min. After cooling to room temperature, the digested samples were transferred to Falcon tubes, adjusted to a final volume of 50 mL with deionized water, and further diluted as necessary for analysis. The analysis-ready samples were loaded into the ICP-MS. Solvent-derived element background was accounted for using a blank. The ICP-MS results, yielded in ppm or ppb, were then converted to Ca and P quantities by applying the appropriate dilution factors. All measurements were performed in triplicate and averaged. Data are reported in mg/g bone. The Ca/P ratio was calculated using the established quantities. The instrument characteristics and operating conditions during analyses are detailed in Appendix A.3.

### 4.5. Statistical Analysis

The Shapiro–Wilk test was conducted to determine whether the data were normally distributed. For normally distributed data, an ANOVA was performed, followed by Fisher’s Least Significant Difference (LSD) pairwise comparisons with Bonferroni correction or Tamhane’s T2 test for multiple comparisons, depending on the homogeneity of variances. For non-normally distributed data, the Kruskal–Wallis H test was used, followed by Dunn’s test with Bonferroni correction for multiple pairwise comparisons. Statistical significance was defined as *p* ≤ 0.05. Analyses were conducted using the statistical packages IBM SPSS Statistics (Release 21.0, IBM Corp., Armonk, NY, USA) and Statistica (Release 12.5, StatSoft Inc., Tulsa, OK, USA) for Windows, as applicable. Figures were generated using GraphPad Prism (Release 10.6.1, GraphPad Software, La Jolla, CA, USA) for Windows. Percentage change was used to express the amount of change between groups for the parameters being compared (Appendix A.4).

After confirming that the assumptions were met, an ANCOVA was conducted to determine whether AAI induction and treatments influenced whole-bone bending properties not explained by CSG. In the ANCOVA, a general linear model was employed in which MU was the dependent variable, SM was the covariate, and group was the fixed factor [38,41]. Pairwise comparisons of adjusted means were performed using Bonferroni correction for multiple group comparisons.

## 5. Conclusions

This study demonstrates that OVA-induced AAI significantly impairs skeletal integrity through the deterioration of bone mechanical properties, geometry, and mineral integrity. Our findings establish a clear hierarchy of therapeutic efficacy: while FP and low-dose anti-IgE offered minimal protection, high-dose anti-IgE outperformed anti-TNF across tissue-level and physicochemical parameters, identifying the IgE-mediated pathway as a primary driver of bone deterioration in this model. Ultimately, the combination of high-dose anti-IgE and FP provided the most robust preservation of the mechanical and mineral integrity of bone. These results underscore the significance of the “lung–bone axis” and suggest that integrated therapies targeting the IgE/mast cell axis are key to safeguarding skeletal health in patients with IgE-mediated, Th2-driven allergic airway inflammation.

## Figures and Tables

**Table 1:** Comparisons of bone cross-sectional geometry features of the groups.

Parameters	Control	AAI	Treatment Groups				
			FP	aIgE-L	aIgE-H	aTNF	FP/aIgE-H
Tibial length (Lo; mm)	18.85 ± 0.32	18.53 ± 0.35	18.55 ± 0.40	18.57 ± 0.49	18.82 ± 0.60	18.72 ± 0.41	18.86 ± 0.58
Span length (L; mm)	13.27 ± 0.22	12.99 ± 0.32	13.00 ± 0.24	13.01 ± 0.29	13.20 ± 0.44	13.19 ± 0.30	13.31 ± 0.21
Aspect ratio (Ar; mm/mm)	6.29 ± 0.17	6.17 ± 0.19	6.06 ± 0.15	6.11 ± 0.13	6.23 ± 0.20	6.21 ± 0.13	6.25 ± 0.19
Cross-sectional cortical area (CSA; mm^2^)	3.64 ± 0.04	3.11 ± 0.06 ^a^*	3.49 ± 0.06	3.60 ± 0.07	3.98 ± 0.07 ^b‡,c†^	3.77 ± 0.10 ^b†^	4.36 ± 0.09 ^a^*^,b‡,c‡,d†^
Cross-sectional moment of inertia (CSMI; mm^4^)	1.29 ± 0.07	0.93 ± 0.07 ^a^*	1.04 ± 0.05	1.27 ± 0.04	1.35 ± 0.07 ^b†,c^*	1.38 ± 0.10 ^b†,c^*	1.42 ± 0.08 ^b‡,c‡^
Section modulus (SM; mm^3^)	1.22 ± 0.09	0.88 ± 0.07 ^a‡^	0.97 ± 0.06 ^a‡^	1.18 ± 0.02 ^b‡,c‡^	1.27 ± 0.08 ^b‡,c‡^	1.30 ± 0.09 ^b‡,c‡^	1.33 ± 0.10 ^b‡,c‡^

*n* = 8 per group. The data are expressed as mean ± SD. One-way analysis of variance (ANOVA), followed by Fisher’s Least Significant Difference (LSD) pairwise comparisons with Bonferroni correction (for Lo) or Tamhane T2 (for L and SM) post hoc test or Kruskal–Wallis H test (for Ar, CSA, and CSMI), followed by Dunn’s post hoc test with Bonferroni correction for multiple comparisons, were used. ^a^ vs. control, ^b^ vs. AAI, ^c^ vs. FP, ^d^ vs. aIgE-L. * *p* < 0.05, ^†^ *p* < 0.01, ^‡^ *p* < 0.001. F (6, 49) = 0.793, *p* = 0.580, and η^2^ = 0.089 for Lo; F (6, 49) = 1.708, *p* = 0.139, and η^2^ = 0.173 for L; H (6, N_Total_ = 56) = 9.71, *p* = 0.137, and $\eta_{H}^{2}$ = 0.177 for Ar; H (6, N_Total_ = 56) = 51.82, *p* = 0.0001, and $\eta_{H}^{2}$ = 0.942 for CSA; H (6, N_Total_ = 56) = 42.25, *p* = 0.0001, and $\eta_{H}^{2}$ = 0.768 for CSMI; F (6, 49) = 41.14, *p* = 0.0001, and η^2^ = 0.834 for SM. Cases with statistical differences are shown. Control (saline); AAI (allergic airway inflammation; OVA-sensitized/challenged); FP (2000 µg fluticasone propionate); aIgE-L (100 µg anti-IgE); aIgE-H (200 µg anti-IgE); aTNF (6.25 mg/kg anti-TNF); FP/aIgE-H (combination of 2000 µg FP/200 µg aIgE). All treatments were administered during the OVA challenge phase.

**Table 2:** Comparisons of bone biomechanical features of the groups.

Parameters	Control	AAI	Treatment Groups				
			FP	aIgE-L	aIgE-H	aTNF	FP/aIgE-H
Yield moment (MY; Nmm)	28.48 ± 2.66	21.78 ± 4.07 ^a†^	20.28 ± 4.63 ^a‡^	20.56 ± 3.06 ^a†^	27.39 ± 2.69 ^b^*^,c†,d†^	25.62 ± 3.33	30.74 ± 3.64 ^b–d‡^
Ultimate moment (MU; Nmm)	32.31 ± 2.79	24.81 ± 4.55 ^a†^	23.36 ± 5.02 ^a‡^	23.71 ± 3.38 ^a†^	31.12 ± 4.50 ^b^*^,c†,d†^	29.22 ± 1.61	35.35 ± 3.10 ^b–d‡^
Rigidity (R; Nmm^2^)	1421 ± 108	1187 ± 88 ^a†^	1195 ± 97 ^a†^	1186 ± 100 ^a†^	1408 ± 109 ^b–d†^	1400 ± 106 ^b–d†^	1533 ± 113 ^b–d‡^
Energy to failure (EtF; N)	1.35 ± 0.14	0.84 ± 0.19 ^a‡^	0.83 ± 0.20 ^a‡^	0.89 ± 0.21 ^a†^	1.45 ± 0.25 ^b–d‡^	1.42 ± 0.18 ^b–d‡^	1.48 ± 0.23 ^b–d‡^
Post-yield displacement (PYD; mm/mm^2^)	0.022 ± 0.006	0.018 ± 0.005	0.016 ± 0.006	0.017 ± 0.005	0.029 ± 0.006 ^c†,d^*	0.028 ± 0.008 ^c^*	0.024 ± 0.007

*n* = 8 per group. The data are expressed as mean ± SD. One-way ANOVA (for MY, MU, R, and EtF), followed by Fisher’s LSD pairwise comparisons with Bonferroni correction, or Kruskal–Wallis H test (for PYD), followed by Dunn’s post hoc test with Bonferroni correction for multiple comparisons, were used. ^a^ vs. Control, ^b^ vs. AAI, ^c^ vs. FP, ^d^ vs. aIgE-L. * *p* < 0.05, ^†^ *p* < 0.01, ^‡^ *p* < 0.001. F (6, 49) = 11.23, *p* = 0.0001, η^2^ = 0.579 for MY; F (6, 49) = 12.61, *p* = 0.0001, η^2^ = 0.607 for MU; F (6, 49) = 14.98, *p* = 0.0001, and η^2^ = 0.647 for R; F (6, 49) = 18.74, *p* = 0.0001, and η^2^ = 0.696 for EtF; H (6, N_Total_ = 56) = 24.13, *p* = 0.0001, and $\eta_{H}^{2}$ = 0.439 for PYD. Cases with statistical differences are shown. Control (saline); AAI (allergic airway inflammation; OVA-sensitized/challenged); FP (2000 µg fluticasone propionate); aIgE-L (100 µg anti-IgE); aIgE-H (200 µg anti-IgE); aTNF (6.25 mg/kg anti-TNF); FP/aIgE-H (combination of 2000 µg FP/200 µg aIgE). All treatments were administered during the OVA challenge phase.

**Table 3:** One-way analysis of covariance (ANCOVA) results of the groups.

ANCOVA (F (6, 48) = 10.288, *p* < 0.0001, η^2^ = 0.563) Controlling for Section Modulus (SM)						
Group (I)	Ultimate Moment (MU; Nmm)		Pairwise Comparisons			
	Mean ± SD	Adjusted Mean ± SE *	Group (J)	Mean Difference (I-J)	SE	Sig.** (*p*-Value)
Control	32.31 ± 2.79	33.24 ± 1.31	AAI	13.224 ***	2.869	0.001
			FP	13.154 ***	2.427	<0.0001
			aIgE-L	9.279 ***	1.792	<0.0001
			aIgE-H	0.280	1.807	1.000
			aTNF	1.791	1.844	1.000
			FP/aIgE-H	−4.914	1.919	0.286
AAI	24.81 ± 4.55	20.02 ± 2.27	Control	−13.224 ***	2.869	0.001
			FP	−0.070	1.870	1.000
			aIgE-L	−3.945	2.663	1.000
			aIgE-H	−12.944 ***	3.159	0.003
			aTNF	−11.434 ***	3.288	0.023
			FP/aIgE-H	−18.138 ***	3.479	<0.0001
FP	23.36 ± 5.02	20.09 ± 1.80	Control	−13.154 ***	2.427	<0.0001
			AAI	0.070	1.870	1.000
			aIgE-L	−3.875	2.252	1.000
			aIgE-H	−12.874 ***	2.685	<0.0001
			aTNF	−11.363 ***	2.802	0.004
			FP/aIgE-H	−18.068 ***	2.979	<0.0001
aIgE-L	23.71 ± 3.38	23.96 ± 1.26	Control	−9.279 ***	1.792	<0.0001
			AAI	3.945	2.663	1.000
			FP	3.875	2.252	1.000
			aIgE-H	−8.999 ***	1.879	<0.0001
			aTNF	−7.489 ***	1.936	0.007
			FP/aIgE-H	−14.193 ***	2.037	<0.0001
aIgE-H	31.12 ± 4.50	32.96 ± 1.45	Control	−0.280	1.807	1.000
			AAI	12.944 ***	3.159	0.003
			FP	12.874 ***	2.685	<0.0001
			aIgE-L	8.999 ***	1.879	<0.0001
			aTNF	1.510	1.778	1.000
			FP/aIgE-H	−5.194	1.811	0.129
aTNF	29.22 ± 1.61	31.45 ± 1.53	Control	−1.791	1.844	1.000
			AAI	11.434 ***	3.288	0.023
			FP	11.363 ***	2.802	0.004
			aIgE-L	7.489 ***	1.936	0.007
			aIgE-H	−1.510	1.778	1.000
			FP/aIgE-H	−6.705 ***	1.786	0.010
FP/aIgE-H	35.35 ± 3.10	38.15 ± 1.67	Control	4.914	1.919	0.286
			AAI	18.138 ***	3.479	<0.0001
			FP	18.068 ***	2.979	<0.0001
			aIgE-L	14.193 ***	2.037	<0.0001
			aIgE-H	5.194	1.811	0.129
			aTNF	6.705 ***	1.786	0.010

SD denotes the standard deviation of the mean. SE denotes the standard error of the mean. * Represents the means of each group once the covariate was controlled. ** Adjustment for multiple comparisons: Bonferroni. *** The mean difference is significant at the 0.05 level. Control (saline); AAI (allergic airway inflammation; OVA-sensitized/challenged); FP (2000 µg fluticasone propionate); aIgE-L (100 µg anti-IgE); aIgE-H (200 µg anti-IgE); aTNF (6.25 mg/kg anti-TNF); FP/aIgE-H (combination of 2000 µg FP/200 µg aIgE). All treatments were administered during the OVA challenge phase.

## Data Availability

The original contributions presented in this study are included in this article/Appendix A. Further inquiries can be directed to the corresponding author.

## Appendix A

### Appendix A.1. Animal Care and Bone Tissue Handling

Male BALB/c mice were acquired from the Gaziantep University Experimental Animals Research Center (GAÜNDAM) and kept under standard conditions (12 h light/dark cycle; temperature, 22 ± 2 °C; relative humidity, 50–70%; standard diet; purified water ad libitum) throughout the study. An acclimation period of 24 h was provided, adhering to the Guidelines for the Housing of Mice in Scientific Institutions for on-site purchase [76].

Bones analyzed in the current study were obtained through secondary harvest from a prior study examining the impact of anti-TNF, FP, and/or anti-IgE on the chronic pulmonary alterations in BALB/c mice with experimentally induced chronic asthma [62]. In the previous study [62], 56 male BALB/c mice were assigned to seven experimental groups using restricted randomization to achieve balanced group sizes (*n* = 8 per group; 56 in total). This method preserved statistical power between groups and reduced the risk of confounding factors that could affect morphometric, histopathological, and bronchoalveolar lavage fluid analyses, such as differences in body weight and age, which can arise from physiological imbalances. The selection of FP and aIgE-L/-H doses was based on their proven efficacy within pharmacologically active preclinical ranges derived from earlier empirical murine models [68,69,70,71,77]. In a similar vein, the administered aTNF dose (6.25 mg/kg i.p.) adhered to the methodological precedent established by Deveci et al. (2008), who adapted this regimen from established protocols for rheumatoid arthritis and Crohn’s disease [72]. A single investigator performed all animal handling, treatment interventions, anesthesia, thoracotomy, and euthanasia to eliminate inter-operator variability in stress responses. A previous study [62] also used a tissue-sharing approach to comply with the reduction principle of the 3Rs (replacement, reduction, and refinement), aiming to maximize the scientific value of each animal and to minimize the total number of animals required for research. Left tibiae were harvested for the present study immediately after the terminal procedure (lung sample removal) of the prior study. No additional animals were used specifically for the present study.

Following collection, the samples were transported within a UN 3373 (Biological Substance, Category B)-compliant insulated medical carrier to the Department of Biophysics (Mersin University Faculty of Medicine), adhering to cold-chain principles. Bone tissues were then stored at −20 °C in a standard high-performance laboratory freezer until the geometric, mechanical and compositional analyses were conducted. The measurements were carried out in three distinct facilities located in the center campus of Mersin University: first, cross-sectional geometry at the Radiodiagnostics Department of the Medical School Hospital, then mechanical analyses at the Biophysics Department, and finally, ICP-MS at the Advanced Technology Education Research and Application Center (MEITAM). On the day of measurements and tests, the samples were thawed once. All measurements and tests were completed on the same day. Experimental conditions were standardized across all three facilities; each respective laboratory environment was climate-controlled to a constant temperature of 22–24 °C. During inter-center transfer, the samples were enveloped in saline-soaked gauze to avoid desiccation and transported within a UN 3373-certified insulated medical carrier. As recommended by Turner and Burr (2001), non-tested samples were maintained in normal saline between or during tests [74]. During the mounting and testing, the bones were routinely moistened with normal saline [78].

### Appendix A.2. Experimental Setup and Conditions for Mechanical Testing

At the macroscopic level, the structural behavior of bone is determined via whole-bone mechanical testing [79]. For this purpose, various bone regions, including the tibia [37,38,54,80,81], and a variety of mechanical techniques, such as tensile [82], four-point [83] and three-point bending [37,38,39,54,80,81,84] tests, have been employed. In the current study, tibial whole-bone mechanical features were assessed via a three-point bending test.

*Preparing tibia bones for mechanical testing*: Before the three-point bending test, the bones were immersed in room-temperature normal saline for 30 min. Prior to testing, each tibia was evaluated for quality according to predetermined inclusion and exclusion criteria. To prevent human factor confounders, a primary investigator, blinded to the group identity of each tibia (samples labeled with unique alphanumeric codes), removed surrounding musculature and conducted visual and microscopic examinations using a standard stereo microscope. Bones were included only if they exhibited no anatomical abnormalities, had confirmed identity, and showed no macroscopic/microscopic fractures or cracks. As no specimens met the exclusion criteria, all 56 tibiae were included in the subsequent analysis. Throughout the assembly and testing, the bones awaiting mechanical testing were kept in Petri dishes filled with normal saline. Since the measured mechanical features were influenced by ambient temperature, it was held at 22–24 °C.

*Experimental conditions*: The same investigator conducted all tests. Blinding of the investigator was maintained throughout data collection and mechanical analysis. For testing, the bone was positioned horizontally on the two lower supports of the device with its medial surface facing upwards. During mounting and testing, the bones were routinely moistened with normal saline to avoid desiccation. Load in all tests was conducted perpendicular to tibial mid-diaphysis in the ML plane with a constant loading speed of 1 mm/s and at a strain rate of 0.061 ± 0.003 s^−1^ until failure. Signals resulting in pressing load were recorded via a 12-bit A/D converter with a sampling rate of 1000 samples/s. Specimens were continuously monitored for technical failures; no slippage or premature fractures were observed.

### Appendix A.3. ICP-MS Device Properties and Working Conditions of the Device During Analyses

*Properties of ICP-MS device*: Brand/Model: Agilent-7500ce (Tokyo, Japan); software: ChemStation (G1834B) (ensures that each sample is safely analyzed and the data obtained is interpreted correctly); system: Octopole reaction system; purity of argon gas used: 99.998%; calibration range: 0-to-90 ppb in 10 ppb steps (calibration was performed using multi-element calibration standards having internationally recognized standards); elemental detection limits: <1 ppb.

*Working conditions of the device during analyses*: RF Power: 1500 W; Sample Dept: 8.8 mm; Plasma Gas: 15 L/min; Carrier Gas: 0.9 L/min; MakeUP Gas: 0.14 L/min; Aux Gas: 1 L/min; Nebulizer Pump: 0.1 rps; S/C Temp: 2 °C; Mod: No gas mod; Integration Time: 0.60 s; Rep: 3; Interference Correction: ON.

### Appendix A.4. Calculation of Percentage of Change

In the present study, in order to express the amount of change between the groups in terms of compared parameters, the percentage change was used. The amount of percentage change was calculated using the following equation:

(A1) $$Percentage\;of\;change=\frac{mean\;of\;the\;final\;group-mean\;of\;the\;initial\;group}{mean\;of\;the\;initial\;group}\times 100$$
